# Recruitment and Activation of Pancreatic Stellate Cells from the Bone Marrow in Pancreatic Cancer: A Model of Tumor-Host Interaction

**DOI:** 10.1371/journal.pone.0026088

**Published:** 2011-10-14

**Authors:** Christopher J. Scarlett, Emily K. Colvin, Mark Pinese, David K. Chang, Adrienne L. Morey, Elizabeth A. Musgrove, Marina Pajic, Minoti Apte, Susan M. Henshall, Robert L. Sutherland, James G. Kench, Andrew V. Biankin

**Affiliations:** 1 Cancer Research Program, Garvan Institute of Medical Research, Darlinghurst, Sydney, Australia; 2 Division of Surgery, Bankstown Hospital, Eldridge Road, Bankstown, Sydney, Australia; 3 Department of Anatomical Pathology, St Vincent's Hospital, Darlinghurst, Australia; 4 South Western Sydney Clinical School, The University of New South Wales, Sydney, Australia; 5 Department of Anatomical Pathology, Royal Prince Alfred Hospital, Camperdown, Sydney, Australia; Technische Universität München, Germany

## Abstract

**Background and Aims:**

Chronic pancreatitis and pancreatic cancer are characterised by extensive stellate cell mediated fibrosis, and current therapeutic development includes targeting pancreatic cancer stroma and tumor-host interactions. Recent evidence has suggested that circulating bone marrow derived stem cells (BMDC) contribute to solid organs. We aimed to define the role of circulating haematopoietic cells in the normal and diseased pancreas.

**Methods:**

Whole bone marrow was harvested from male β-actin-EGFP donor mice and transplanted into irradiated female recipient C57/BL6 mice. Chronic pancreatitis was induced with repeat injections of caerulein, while carcinogenesis was induced with an intrapancreatic injection of dimethylbenzanthracene (DMBA). Phenotype of engrafted donor-derived cells within the pancreas was assessed by immunohistochemistry, immunofluorescence and *in situ* hybridisation.

**Results:**

GFP positive cells were visible in the exocrine pancreatic epithelia from 3 months post transplantation. These exhibited acinar morphology and were positive for amylase and peanut agglutinin. Mice administered caerulein developed chronic pancreatitis while DMBA mice exhibited precursor lesions and pancreatic cancer. No acinar cells were identified to be donor-derived upon cessation of cerulein treatment, however rare occurrences of bone marrow-derived acinar cells were observed during pancreatic regeneration. Increased recruitment of BMDC was observed within the desmoplastic stroma, contributing to the activated pancreatic stellate cell (PaSC) population in both diseases. Expression of stellate cell markers CELSR3, PBX1 and GFAP was observed in BMD cancer-associated PaSCs, however cancer-associated, but not pancreatitis-associated BMD PaSCs, expressed the cancer PaSC specific marker CELSR3.

**Conclusions:**

This study demonstrates that BMDC can incorporate into the pancreas and adopt the differentiated state of the exocrine compartment. BMDC that contribute to the activated PaSC population in chronic pancreatitis and pancreatic cancer have different phenotypes, and may play important roles in these diseases. Further, bone marrow transplantation may provide a useful model for the study of tumor-host interactions in cancer and pancreatitis.

## Introduction

Pancreatic cancer (PC) remains one of the most devastating cancers, and is the fourth leading cause of cancer death in western societies with a survival rate of less than 5% [1]. Nothing apart from pancreatic resection in a proportion of patients (10–20%), offers any curative potential, with chemotherapeutic agents meeting limited success [2]. Chronic pancreatitis is a significant risk factor for the development of pancreatic cancer and both are characterised by extensive stellate cell mediated fibrosis, which in the case of pancreatic cancer facilitates cancer progression and metastasis [3], [4]. Recently, Olive *et al* [5] demonstrated that, by targeting the stroma using inhibitors of hedgehog signalling, significantly improves the delivery of chemotherapeutic agents to the epithelial compartment of the tumor, and although the effect was transient, improved overall efficacy. Further, Kraman *et al* demonstrated that targeting specific sub-populations of stromal cells for destruction could remove their inhibitory effect on the host's immune response to the tumor [6].

Observations made in recent years have demonstrated that adult stem cells have remarkable flexibility in their differentiation repertoires. This plasticity allows adult stem cells, particularly those of bone marrow origin, to engraft alternative non-haematopoietic locations and transdifferentiate into cell types appropriate to their new niche. This is particularly evident when the recipient organ is damaged [7], [8]. Bone marrow derived cells (BMDC) can either engraft or fuse to adopt, or be reprogrammed, to the differentiated state of the particular epithelia [9] (reviewed in [10]). This suggests that the endogenous stem cell of an organ, and its role in growth and regeneration, is not confined to each specific organ but may be a dynamic system involving circulating BMDC with stem cell niche environments regulating recruitment, proliferation and differentiation [7], [11]. This may have significant implications concerning the evolution of cancers in many solid organs, including the pancreas. Houghton *et al* demonstrated that in a model of *Helicobacter felis* induced gastric carcinogenesis, the development of metaplasia and dysplasia was linked to the engraftment and expansion of the BMDC population, eventually giving rise to gastric adenocarcinoma [12]. Observations in women who received bone marrow transplants from male donors, and who subsequently developed a cancer, identified that myofibroblasts (pancreatic stellate cell equivalent) within these tumors were derived from donor bone marrow [13].

The majority of previous studies assessing the role of BMDC in pancreatic regeneration and repair have concentrated on restoring endocrine function following islet cell injury [14], [15], [16], [17], [18], [19], [20], [21]. Few studies have focussed on the contribution of BMDC to growth and regeneration of the exocrine pancreas, or their role in pancreatic cancer. Wang *et al* [22] describe the contribution of BMDC to pancreatic duct formation in neonatal mice, Marrache *et al* [23], and Watanabe *et al* [24] demonstrate in a model of caerulein induced chronic pancreatitis that BMDC contribute to the pancreatic stellate cell population suggesting a role in tissue repair, while more recently Pan *et al* [25] identified a contribution of BMDC to the pancreatic stellate cell population in a rat model of chemical carcinogenesis.

Here we generate a robust model of whole bone marrow transplantation to show that in pancreatic carcinogenesis, and in chronic pancreatitis, BMDC contribute significantly to the activated pancreatic stellate cell (PaSC) population. Those associated with pancreatic cancer express genes **characteristic of peritumoral stellate cells as compared to those not associated with malignancy**, suggesting that BMDC may play an important role in supporting pancreatic carcinogenesis. In addition, these models of bone marrow transplantation may prove useful in investigating tumor-host interactions *in vivo*.

## Methods

### Ethics Statement

All animal work was approved by the Garvan Institute of Medical Research/St Vincent's Hospital Animal Ethics Committee (Protocol #06/53).

### Bone Marrow Transplantation

Whole bone marrow was harvested from male C57/BL6-TgN(ACTbEGFP)IOsb/J (The Jackson Laboratory, Bar Harbor, ME, USA; hereafter referred to as the β-actin-EGFP mouse) by flushing the tibias and femurs with Dulbecco's Modified Eagle's Medium (DMEM; Invitrogen, Eugene, OR, USA) using a 26G needle. Cells were filtered through a 70 µM cell strainer, counted, then washed and resuspended in PBS. 5×10^6^ β-actin-EGFP bone marrow cells were transplanted into irradiated (950 Rads; Gammacell 40 Exactor; Nordion International Inc. Canada) recipient 4–8 week old female C57/B6 mice via the tail vein. Mice were given antibiotic water (40 mg/5 ml trimethoprim + 200 mg/5 ml sulfamethoxazole) for 14 days. While the use of enhanced green fluorescent protein (GFP) has become the marker of choice for many types of cell transplantation and lineage marking experiments, it is not clear that the GFP expressed in bone marrow stem cells would continue to be expressed in non-haematopoietic tissues following nuclear reprogramming [26], [27], [28]. Consequently, we performed gender mismatched transplants to track the fate of donor-derived cells using the Y chromosome genotypic marker to validate the findings observed using the GFP reporter.

### Normal Pancreas

**Figure 1:**
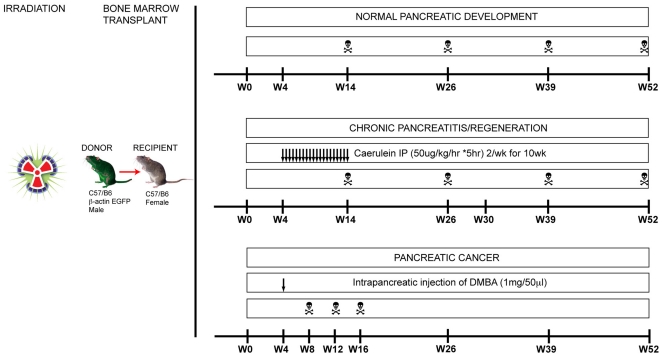
Experimental overview. 5×10^6^ bone marrow cells from male β-actin-EGFP mice were transplanted into lethally irradiated female C57/B6 mice. For assessment of the normal pancreas, pancreata were harvested at 3, 6, 9 and 12 months post transplant. For chronic pancreatitis, mice were administered caerulein intraperitoneally for 10 weeks then sacrificed upon cessation of the treatment, as well as 3, 6 and 9 months post treatment to assess regeneration. For pancreatic cancer, at 1 month post transplant mice were administered an intrapancreatic injection of 7,12-dimethylbenzanthracene (DMBA) and sacrificed by 4, 8, 12 or >12 months post DMBA treatment or when a pancreatic mass was detected.

Mice were sacrificed at 3 (n = 12), 6 (n = 12), 9 (n = 12) and 12 (n = 11) months post transplantation (Figure 1). At each time point, the pancreas was harvested, halved and placed in either 10% neutral buffered formalin then embedded in paraffin, or embedded in cold Tissue-Tek OCT Compound (Sakura Finetek, Torrance, CA, USA) and snap frozen in liquid N_2_. Cellular phenotype of donor derived cells within the pancreas were assessed by immunohistochemistry, immunofluorescence and *in situ* hybridisation for the Y chromosome. To monitor engraftment of donor β-actin-EGFP bone marrow, peripheral blood was assessed for GFP positivity using Fluorescence Activated Cell Sorting (FACS) at 1 month post transplantation, then upon sacrifice (Table 1).

**Table 1:** Assessment of engraftment of donor derived cells in peripheral blood and bone marrow.

	% GFP Positive Cells (95% CI)	
Months post transplant	Peripheral Blood	Bone Marrow
**1**	81.92 (80.71-83.10)	---
**3**	76.26 (71.89-80.37)	25.39 (23.32-27.52)
**6**	86.80 (82.77-90.37)	22.26 (19.63-25.01)
**9**	73.81 (68.42-78.85)	22.77 (20.26-25.39)
**12**	83.68 (78.56-88.21)	27.54 (24.83-30.34)

### Chronic Pancreatitis

One month after transplantation, mice were injected intraperitoneally with 50 µg/kg of caerulein (#152860; MP Biomedicals Inc., Solon, OH, USA) 5 times over 4 consecutive hours, twice a week for 10 weeks as previously described [29]. Mice were sacrificed upon completion of caerulein treatment (n = 8) and 3 (n = 8), 6 (n = 8) and 9 (n = 8) months post caerulein treatment to assess pancreatic regeneration. An additional arm assessed pancreatic regeneration in mice treated with caerulein at 6 months post transplantation and sacrificed at 3 months post cessation of caerulein treatment (n = 4) (Figure 1).

### Chemical Model of Pancreatic Cancer

One month after transplantation, mice were anaesthetised with isofluorane and a left lateral subcostal incision in the abdominal wall was performed and the spleen exteriorised to reveal the body and tail of the pancreas. 1 mg/50 µl of 7,12-dimethylbenzanthracene (DMBA; Sigma-Aldrich, St Louis, MO, USA) was dissolved in PBS/0.1% TWEEN and injected into the tail of the pancreas using a 25G needle as previously described [30]. The spleen and pancreas were returned to the abdominal cavity and the peritoneum/muscle layers and skin were sutured closed. Mice were sacrificed between 1–4 (n = 16), 5–8 (n = 6), 9–12 (n = 4) and >12 (n = 2) weeks post DMBA treatment or when a pancreatic mass was detected following abdominal palpation (Figure 1). As with the model of chronic pancreatitis, an additional arm assessed the contribution of BMDC to pancreatic carcinogenesis following induction with DMBA at both 6 (n = 10) and 9 (n = 10) months post transplant.

### FACS analysis

Peripheral blood and bone marrow cells were analysed for GFP expression using fluorescence-activated cell sorting (FACS) (Table 1).

#### Peripheral Blood

At 1 month post transplantation and upon sacrifice, blood was collected via tail bleed and collected in BD Microtainers with lithium/heparin (BD, Franklin Lakes, NJ, USA). 1000 µl FACSlyse (1X) was then added and incubated for 10 minutes at room temperature. The tubes were centrifuged at 1000 g for 5 mins, the supernatant removed and the cells resuspended in 200 µl of cold PBS on ice until analysis.

#### Bone Marrow

Bone marrow was flushed from the femurs of transplanted mice with cold PBS using a 26G needle, filtered through a 70 µM cell strainer and held on ice until analysis. Fluorescence-activated cell sorting (FACS) analysis was carried out using a BD FACS CANTO (BD, Franklin Lakes, NJ, USA).

### Immunohistochemistry/Immunofluorescence/In-situ hybridisation

#### Immunohistochemistry

Paraffin embedded pancreatic tissue sections (4 µm) were de-waxed and rehydrated before H&E staining to assess histological morphology. For immunohistochemistry, antigen unmasking was achieved using target-retrieval solution (s2367, pH 9.0: DAKO Corporation, Carpenteria, California, USA) for 30 minutes in a boiling water bath. Endogenous peroxidase activity was quenched with 3% hydrogen peroxide (5 minutes), rinsed in DAKO buffer (DAKO Corporation) then slides were blocked with DAKO Protein block (10 minutes; DAKO Corporation). Sections were then incubated for 60 minutes with primary antibody (see below) then a labelled polymer horseradish peroxidase anti-rabbit detection system was used (30 minutes; Envision+ anti-rabbit; DAKO Corporation) and 3,3′-diaminobenzidine was used as a substrate. Counter-staining was performed with Mayer's hematoxylin (DAKO Corporation).

#### Immunofluorescence

For immunofluorescence analysis, 4 µm paraffin sections were de-waxed, rehydrated and antigen retrieved as described above. Sections were then blocked with Protein block (10 minutes), then primary antibodies (see below) were incubated for 1 hour at room temperature, washed in DAKO buffer (3×5 minutes) and incubated in secondary antibodies (see below) for 1 hour at room temperature. Fluorescent staining of apical membranes of exocrine acinar cells was performed using rhodamine-conjugated peanut agglutinin (PNA; 1∶200; Vector Laboratories, Burlingame, CA, USA). The slides were rinsed then counterstained and mounted in Vectashield hard set mounting medium with DAPI (H-1500; Vector Laboratories).

Primary Antibodies: GFP rabbit polyclonal (A11122; Invitrogen, Eugene, OR, USA; 1∶1000 IHC, 1∶200 IF), GFP goat polyclonal (ab5450; Abcam, Cambridge, MA, USA; 1∶1000), amylase goat polyclonal (C-20; Santa Cruz Biotechnology Inc., Santa Cruz, CA, USA; 1∶200 IF), desmin (Thermo Scientific, Fremont, CA, USA; 1∶200 IHC), α-Smooth Muscle Actin mouse monoclonal (clone 1A4; A5228; Sigma-Aldrich, St Louis, MO, USA; 1: 1∶500 IHC/IF), Vimentin (SP-20; Epitomics, Burlingame, CA, USA; 1∶200 IHC), Cytokeratin 5/6 (D-13; Santa Cruz Biotechnology Inc., Santa Cruz, CA, USA; 1∶100 IHC), glial fibrillary acidic protein (GFAP) rabbit polyclonal (Z0334; DAKO Corporation, Carpenteria, California, USA; 1∶50), pre-B-cell leukemia transcription factor 1 (PBX1) rabbit polyclonal (ab12001; Abcam, Cambridge, MA, USA; 1∶100), cadherin EGF LAG seven-pass G-type receptor 3 (CELSR3) rabbit polyclonal (ab12958; Abcam, Cambridge, MA, USA; 1∶100).

Secondary Antibodies: anti-rabbit Cy5 (1∶250; #711-176-152; Jackson ImmunoResearch, West Grove, PA, USA), anti-goat Cy5 (1∶250; #705-175-003; Jackson ImmunoResearch, West Grove, PA, USA), anti-goat Cy3 (1∶500; #715-166-147; Jackson ImmunoResearch), anti-mouse Cy3 (1∶500; #715-166-150; Jackson ImmunoResearch).

#### In-situ hybridization

Whole mouse Y chromosome probe labelled with fluorescein (Star*FISH, #1189-YMF-01; Cambio Ltd) was applied to 3 um paraffin sections following deparaffinization in xylene, pre-treatment with DAKO target retrieval solution (TRS: 30 mins at 95°C), protease digestion at 37°C for 15 minutes and post-fixation in neutral buffered formalin for 10 mins. Hybridization site was selected by reference to a serial section stained with H+E. 3.5 µL probe was applied under a 15×15 mm coverslip sealed with rubber cement. Co-denaturation at 90°C was followed by overnight hybridization at 37°C on a thermal cycler block. After post hybridization rinses in 2X SSC/0.3% NP-40 (2×2 mins at 72°C), slides were dried and counterstained with DAPI. Signal was analysed with a Zeiss Axioscope II microscope with digital AxioCam and AxioVision software.

## Results

Successful reconstitution of bone marrow in transplanted mice was demonstrable in peripheral blood at 1 month post transplant using FACS analysis for GFP expression. In addition, long term engraftment was verified at the time of sacrifice. GFP positivity of peripheral blood and bone marrow at each timepoint was comparable to that of the donor β-actin-EGFP mouse (Blood, 70.07%±3.90 SEM; Marrow, 30.87%±1.60 SEM) (Table 1).

### BMDC and normal exocrine pancreas

**Figure 2:**
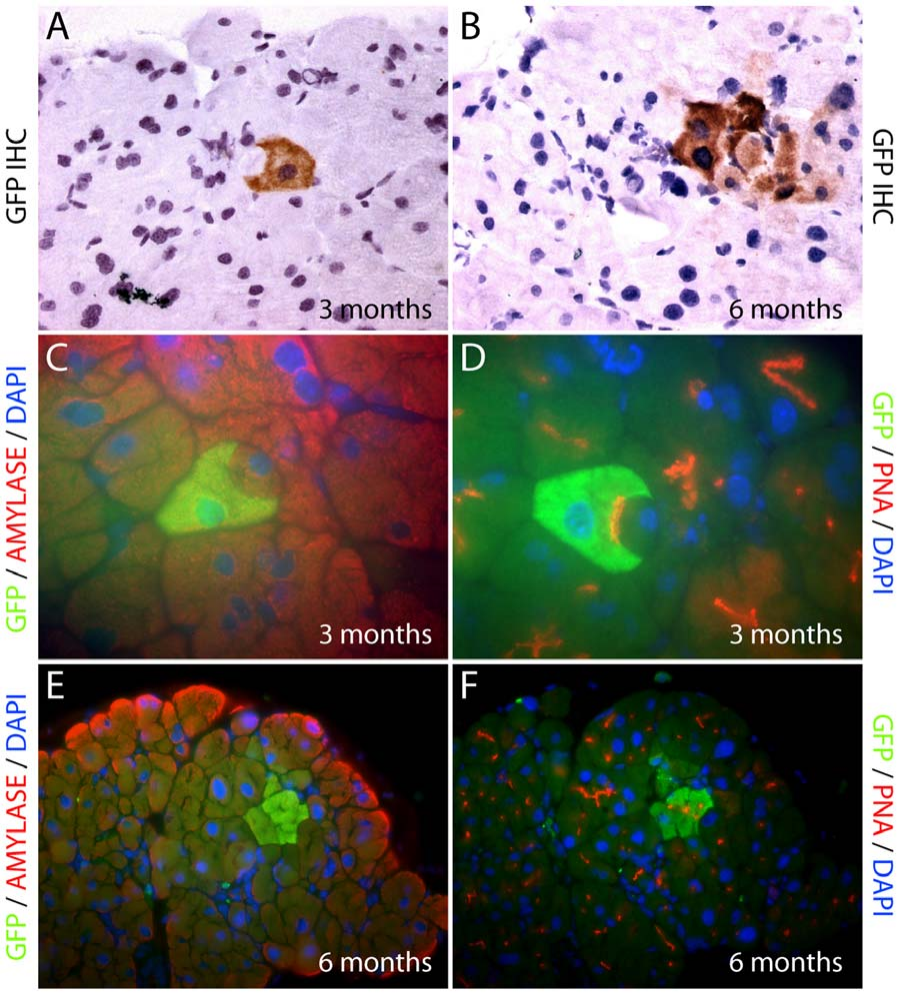
Normal pancreas in bone marrow transplant recipients. GFP immunohistochemistry of pancreata identified individual GFP+ve donor-derived cells with acinar cell morphology from 3 months post transplantation (A), and occasional entire acinar units (B), were observed from 6 months post transplantation. Immunofluorescence images of donor derived GFP+ve acinar cells co-staining positive for both GFP and amylase (C, E); and GFP and PNA (D, F).

From 3 months post transplantation, individual, donor-derived GFP positive cells were seen within the exocrine pancreatic epithelial compartment of non-treated control transplanted mice (Figure 2A). Small GFP positive cells that had spindle-like, or immune cell morphology were scattered throughout the interstitium (data not shown), while rare occurrences of GFP positive cells with acinar morphology were present in the exocrine compartment. Upon further examination using co-immunofluorescence, these cells were positive for GFP, amylase and peanut agglutinin (Figure 2C, 2D), markers specific for pancreatic acinar cells, suggesting that BMDC can incorporate into the adult pancreas and adopt the differentiated state of the exocrine compartment. Cohesive clusters of 2–3 cells were observed from 6 months post transplantation, while occasional entire acinar units consisting of donor-derived GFP positive cells were also evident (Figure 2B, 2E, 2F).

### BMDC and pancreatic injury

An experimental arm to assess engraftment of donor BMDC in pancreas specific injury (chronic pancreatitis) and regeneration was also established. Following 10 weeks of caerulein treatment, mice had pancreata that had atrophied, and peri-acinar fibrosis developed as a fine meshwork throughout the exocrine gland. Intra-acinar lumina were dilated. Some acinar units appeared to develop a ductal phenotype with loss of zymogen granules and more centrally located nuclei. These tubular complexes consisted of cylinders with a wide lumen lined by a monolayer of flattened duct-like cells (Figures S1B, E) [31], [32]. Control pancreata were histologically normal (Figure S1A). Sirius red staining of interstitial collagen was more prominent in the caerulein treated mice, indicative of chronic injury. Staining in control animals was localised primarily around ducts, small blood vessels with thin strands extending between larger lobules, and not around individual acinar units. In the caerulein treated animals periacinar collagen increased, surrounding individual acinar units, similar to the fibrosis observed in human chronic pancreatitis (Figures S1D, E).

**Figure 3:**
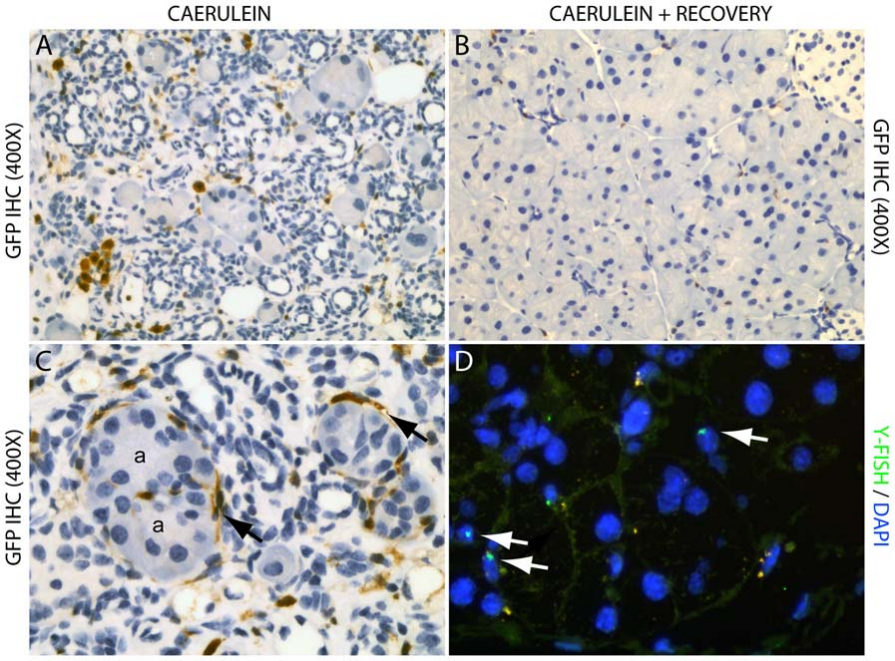
GFP immunohistochemistry of caerulein treated transplanted mice upon cessation of treatment to assess BMDC contribution to pancreatic injury (A), and following 6 months of recovery to assess pancreatic regeneration (B). Note the increased presence of GFP positive cells within the interstitium of the injured pancreas, including inflammatory infiltrate (A), when compared to the regenerated pancreas (B); as well as the GFP positive spindle-like cells (black arrows) with a pancreatic stellate cell morphology (C) surrounding acinar units (a); (D) Y chromosome FISH confirmed the minimal contribution of BMDC to the regeneration of exocrine parenchyma, while some Y positive lymphocytes were observed throughout the parenchyma (white arrows).

Upon cessation of caerulein treatment (3 months post transplantation immediately following injury) there was increased BMDC recruitment to the pancreas (Figure 3A). This included recruitment of GFP positive inflammatory cells such as lymphocytes (positive for CD45 expression; data not shown) and macrophages, while some BMDC located interstitially were positive for desmin, and spindle-like peri-acinar cells possessed the morphology of pancreatic stellate cells (PaSC's; Figure 3C). No acinar cells were identified to be donor derived (GFP positive) at this early timepoint.

### BMDC and pancreatic regeneration

Upon cessation of caerulein treatment, animals were left to recover to assess the contribution of BMDC to pancreatic regeneration. Following 3 months of recovery post cessation of caerulein treatment, pancreata of treated animals returned to a histologically normal phenotype (Figure S1C), resembling that of non-treated animals. A reduction of interstitial and periacinar collagen was also visible (Figure S1F). As for normal pancreas, only rare occurrences of single and small clusters of GFP positive acinar cells displaying amylase and PNA positivity were observed, suggesting that BMDC could adopt the differentiated state of the exocrine compartment during pancreatic regeneration. Interstitial GFP positive inflammatory cells were diminished in number as regeneration time increased (Figure 3B). Similar results were observed for mice that were treated with caerulein following long term engraftment (6 months post transplant) and left to recover. *In situ* hybridisation for the Y chromosome confirmed that there was no increase in the contribution of BMDC to the exocrine pancreas with regeneration (Figure 3D).

### BMDC and pancreatic cancer

Two weeks after injection of DMBA, tubular complexes were present focally among acinar cells with ductal metaplasia adjacent to normal acinar tissue observed by 1 month. From 1 month after DMBA treatment, pancreatic precursor lesions (mPanIN) with varying degrees of dysplasia were present. Foci of adenocarcinoma in relation to mPanIN were seen in the pancreas from 2 months after DMBA, while ductal adenocarcinoma (sarcomatoid variant) developed at 3–4 months (Figures S1G-I). This phenotype closely resembled that seen in a similar model used by Kimura et al [30].

**Figure 4:**
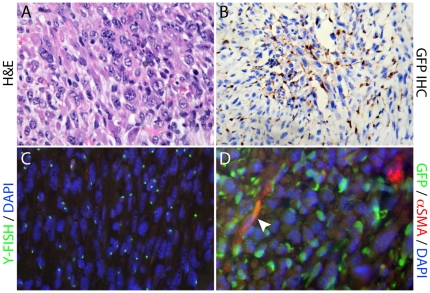
Assessment of the sarcomatoid-like tumor following treatment with DMBA. (A) H&E showing the poorly differentiated, invasive pancreatic tumor; (B) GFP IHC and (C) Y chromosome FISH, demonstrate the extensive bone marrow derived cell population within the tumor; (D) Co-immunofluorescence of GFP with αSMA identified bone marrow derived activated stellate cells within the tumor (arrowhead).

We assessed the contribution of BMDC to the desmoplastic stroma, in particular to the population of pancreatic stellate cells, by assessing co-expression of GFP and the stellate cell selective markers desmin, glial fibrillary acidic protein (GFAP), α-smooth muscle actin (αSMA), the co-expression of which defines activated stellate cells [3], [33], [34] (reviewed in [35]). These markers, originally identified as PaSC specific, are used to distinguish PaSC's from normal fibroblasts due to the co-expression of the intermediate filament proteins desmin and GFAP [33], [34], while expression of αSMA in PaSC's was originally described as a source of fibrosis in chronic pancreatitis and pancreatic cancer, designating activated PaSC's [3], [36]. There was significant BMDC recruitment to the stroma surrounding precursor lesions following DMBA treatment, with contribution of BMDC to the activated pancreatic stellate cell population (GFP, desmin and αSMA positive; Figure S2A,C,E). Co-immunofluorescence analysis also demonstrated that donor-derived BMDC positive for both GFP and αSMA was present in cells directly adjacent to pancreatic intraepithelial neoplasia (mPanIN) lesions (Figure S2B,D,F). We also observed the development of a large, poorly differentiated and invasive cytokeratin positive/vimentin negative ductal adenocarcinoma (sarcomatoid) tumor with a significant BMDC population (Figure 4A, 4B), which included an extensive donor derived inflammatory infiltrate, demonstrated using FISH for the Y-chromosome (Y-FISH; Figure 4C), as well as bone marrow derived activated pancreatic stellate cells (Figure 4D), visualized by co-immunofluorescence of GFP with αSMA (Figure 4D). Bone marrow derived epithelial tumor cells were not seen. We quantified the proportion of BMDC within the tumor microenvironment in 5 high power magnification fields (400X) and determined that 41.8% (± 2.77 SEM) of cells were bone marrow derived (Figure 4). Similar results were observed when we administered DMBA following long term engraftment (6 months post transplant).

More recently, Erkan *et al* [37] used transcript profiling to identify markers to differentiate PaSC’s associated with chronic pancreatitis versus those of pancreatic cancer, with the aim of subtyping PaSC's into either inflammation or cancer-associated. Pre-B-cell leukemia transcription factor 1 (PBX1) was upregulated in inflammation-associated PaSC's compared to tumor-associated PaSC's, while cadherin EGF LAG seven-pass G-type receptor 3 (CELSR3) expression was upregulated in tumor-associated PaSC's compared to that of inflammation-associated PaSC's [37]. We examined 5 sections each from 3 mice that developed adenocarcinoma and observed that GFAP, PBX1 and CELSR3 expression was co-localised with GFP in tumor associated PaSC's (Figure S3A, S3B, S3C respectively), and although PBX1 (but not CELSR3) was detected in the stroma in pancreatitis, it did not co-localise with GFAP, suggesting that it was not expressed in activated stellate cells.

## Discussion

Gender-mismatched whole bone marrow transplants demonstrated that BMDC migrate to the pancreas and expand as clonal units to adopt differentiated states of the exocrine compartment. Although there was minimal recruitment to the acinar cell population, which did not increase with injury, regeneration, or carcinogenesis, there was significant recruitment to the stroma. Although the majority of recruited cells during acute injury and cancer were inflammatory cells, a proportion differentiated into stellate cells. Those associated with cancer expressed markers characteristic of tumor-associated stellate cells suggesting they were co-opted, and altered by the tumor microenvironment.

Whilst regenerative capacity is maintained in the adult exocrine pancreas, the origin of regenerating epithelium remains unclear [38]. Recently, a mechanism of clonal development of pancreatic acini has been described with evidence provided that a single progenitor cell, whether it be a mature acinar cell or a multipotent stem cell, gives rise to all exocrine cells of one acinus [39]. Further, lineage tracing studies provide evidence that new acinar cells are generated from pre-existing acinar cells following partial pancreatectomy [40] and caerulein induced pancreatitis [41]. Given these data, and our observations in the normal pancreas, we hypothesized that BMDCs contribute to the regeneration of the exocrine compartment. Despite using different injury protocol time-points we were unable to demonstrate a significant increase in the BMDC contribution to the acinar cell population. Y chromosome FISH data excluded GFP silencing [42], [43], [44] as a potential cause for lack of GFP expression, and reflected GFP positive cell distribution.

PaSC's are resident myofibroblast-like cells existing in the periacinar space of the exocrine pancreas, and there is increasing evidence to suggest that they are key participants in the pathogenesis of pancreatic exocrine diseases, particularly in the production of abundant fibrous stroma, which is a feature of pancreatic cancer [3]. Although there was significant BMDC recruitment to the inflammatory infiltrate at the time of pancreatic injury consistent with previous reports [45], [46], this was transient and cell numbers diminished over time to low levels when regeneration was complete. However, stellate cells remained amongst the residual population of BMDC. This observation suggests that BMDC play a role in supporting the regenerative process, but do not transform to contribute to the regenerative epithelium itself. Tumor associated BMDC PaSC's may be retained in the peri-tumoral stroma, whilst those associated with pancreatitis are not. Although the number of bone marrow derived PaSC was slightly less in the setting of pancreatitis compared to cancer, longer timepoints would be required to determine if BMDC were preferentially retained in the pancreas in the setting of cancer.

As in chronic pancreatitis, there was increased recruitment of BMDC to the pancreas following treatment with DMBA. This again included an inflammatory infiltrate and activated PaSC's. Importantly, expression of CELSR3 in BMDC associated with tumor suggests that there was modification of these PaSC's by the tumor microenvironment. This is supported by recent studies where bone marrow-derived mesenchymal stem cells preferentially localise to regions of pancreatic tumor growth [47] and have been shown to transform into tumor-associated myofibroblasts in insulinomas [48]. Pancreatic cancer cells secrete growth factors such as TGF-β1, PDGF and VEGF, as well as extracellular matrix (ECM) metalloproteinase inducers that transform the usually quiescent PaSC's into an activated myofibroblast-type phenotype and secrete excess amounts of ECM and matrix degrading enzymes [3], [4], [49] (reviewed in [50]). Vonlaufen and colleagues [51] provide evidence of a bi-directional interaction between tumor cells and pancreatic stellate cells. In addition, orthotopic xenograft models of pancreatic cancer demonstrate that tumors consisting of a mix of tumor cells and PaSC's are larger and exhibit distant regional metastases, with activated PaSC's present within the metastases [51]. The importance of specific stromal cells in tumorigenesis was further defined through a recent study by Kraman *et al* [6], who demonstrated that a sub-population of stromal cells that express fibroblast activation protein (FAP) suppress the immune response and that abrogation of FAP expression arrests the growth of pancreatic tumors, potentially by removing their inhibitory effect on the host's immune response. As a consequence, solid tumor stromal cells, such as PaSC's, present a potential novel therapeutic target [6], [52]. In addition, the presence of activated BMDC PaSC's associated with mPanIN lesions suggest that this process occurs early in pancreatic carcinogenesis.

In conclusion, whilst most cancer associated activated PaSC's are thought to arise from endogenous quiescent PaSC's, we provide evidence that a proportion of these are bone marrow derived, and display different phenotypes depending on whether they are recruited to an inflammatory or a carcinogenic pancreas. Based on evidence identifying cross-talk between pancreatic tumor cells and PaSC's [51], our data suggest that bone marrow-derived PaSC's may play an important, and supportive role in promoting carcinogenesis, the mechanisms of which remain to be elucidated. In addition, these models can potentially be used to selectively manipulate the genetic composition of PaSC's, to facilitate the *in vivo* investigation of tumor-host interaction, where such models have not previously existed.

## Supporting Information

Figure S1 Representative images of the pancreata from control (A, D), caerulein (B–C, E–F) and DMBA treated mice (G–I). Control pancreata were histologically normal, with tightly packed acinar units (A), and interstitial collagen localised primarily around ducts and between large lobules (A, D; arrow). Treatment with caerulein revealed dilated intra-acinar lumina (L) the development of a ductal-like phenotype, presenting as tubular complexes (T), and periacinar fibrosis with increased inflammatory infiltrate (H & B). Sirius red staining revealed increased interstitial (arrow) and periacinar (arrowhead) collagen (E). Following 3 months of recovery post cessation of caerulein, the pancreata returned to a histologically normal phenotype (C) with reduction of interstitial (arrow) and periacinar collagen (F). Treatment with DMBA resulted in the development of mPanIN lesions (*) and pancreatic cancer, which were predominantly mPanIN-1A (G), mPanIN-1B (H), through to the sarcomatoid-like ductal adenocarcinoma (I).
(TIF)

Figure S2 **Immunohistochemical and immunofluorescent analysis of the desmoplastic stroma following transplantation and DMBA treatment.** Serial sections demonstrate positive co-immunohistochemical staining for GFP (A), desmin (C) and αSMA (E). Boxes outline the same individual cells across the serial sections. Co-immunofluorescence for GFP (B) and αSMA (D) showing a bone marrow derived activated pancreatic stellate cell in the stroma directly adjacent to the PanIN lesion.
(TIF)

Figure S3 **Immunofluorescent characterisation of the bone marrow derived stellate cells within the sarcomatoid-like tumor following treatment with DMBA.** Co-immunofluorescence of GFP with (A) glial fibrillary acidic protein (GFAP), (B) pre-B-cell leukemia transcription factor 1 (PBX1), and (C) cadherin EGF LAG seven-pass G-type receptor 3 (CELSR3) to identify bone marrow derived activated stellate cells within the tumor (arrowhead).
(TIF)
