# Supplementary figures and images for: Recruitment and Activation of Pancreatic Stellate Cells from the Bone Marrow in Pancreatic Cancer: A Model of Tumor-Host Interaction
Source: PLoS One. 2011 Oct 14;6(10):e26088. doi: 10.1371/journal.pone.0026088 (PMC3193536; doi:10.1371/journal.pone.0026088)

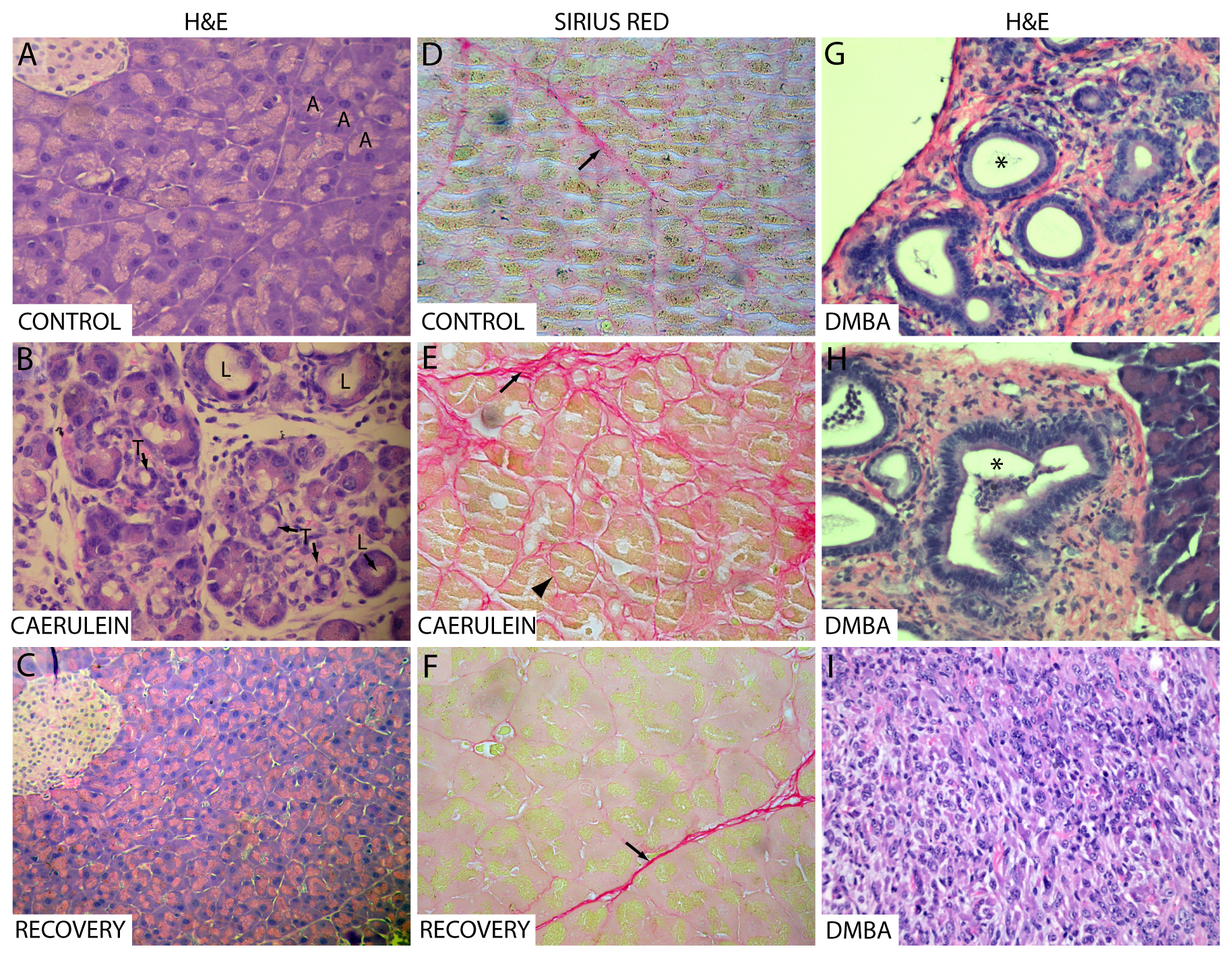

Supplement: Figure S1 — Representative images of the pancreata from control (A, D), caerulein (B–C, E–F) and DMBA treated mice (G–I). Control pancreata were histologically normal, with tightly packed acinar units (A), and interstitial collagen localised primarily around ducts and between large lobules (A, D; arrow). Treatment with caerulein revealed dilated intra-acinar lumina (L) the development of a ductal-like phenotype, presenting as tubular complexes (T), and periacinar fibrosis with increased inflammatory infiltrate (H & B). Sirius red staining revealed increased interstitial (arrow) and periacinar (arrowhead) collagen (E). Following 3 months of recovery post cessation of caerulein, the pancreata returned to a histologically normal phenotype (C) with reduction of interstitial (arrow) and periacinar collagen (F). Treatment with DMBA resulted in the development of mPanIN lesions (*) and pancreatic cancer, which were predominantly mPanIN-1A (G), mPanIN-1B (H), through to the sarcomatoid-like ductal adenocarcinoma (I). (TIF) [file pone.0026088.s001.tif]

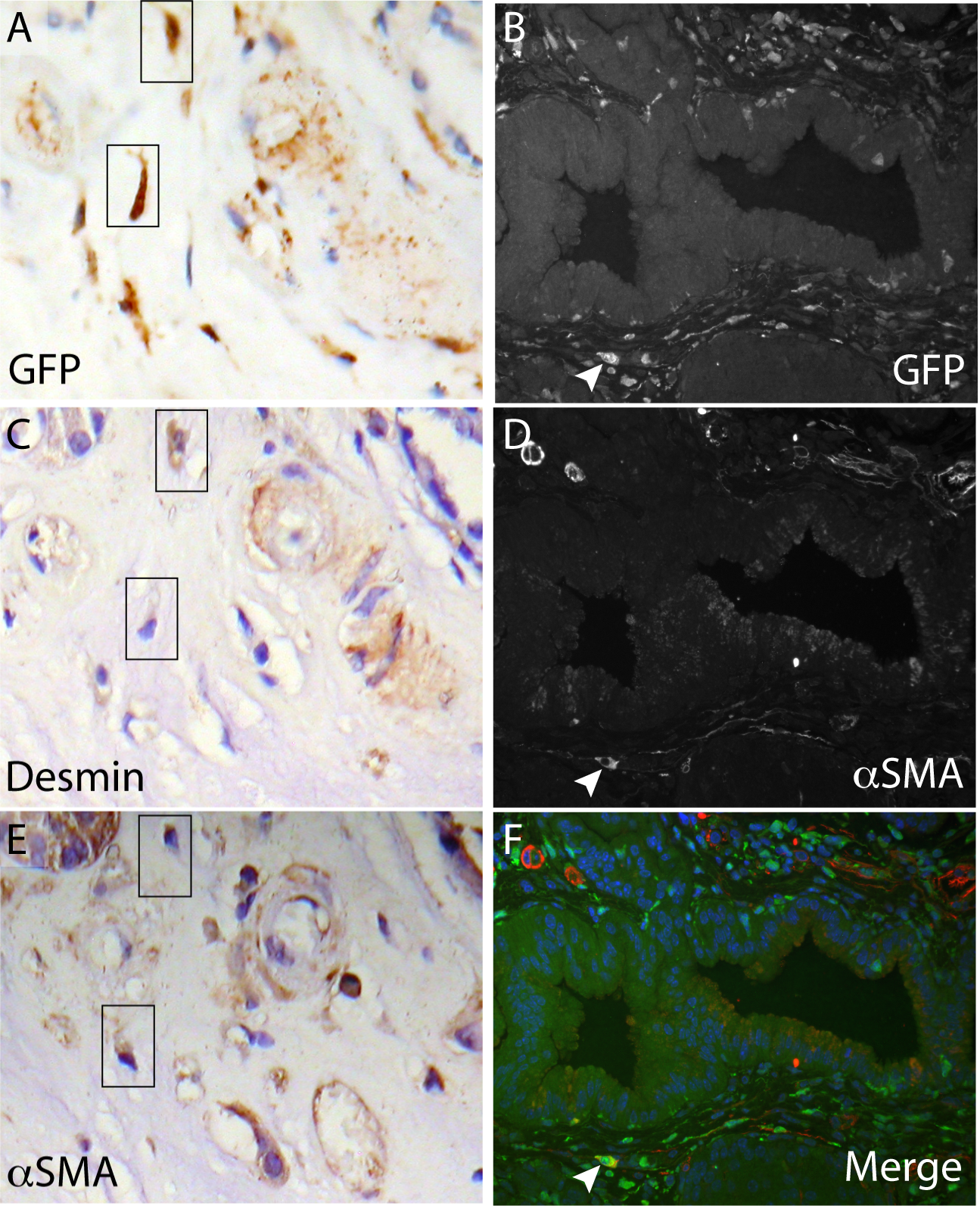

Supplement: Figure S2 — Immunohistochemical and immunofluorescent analysis of the desmoplastic stroma following transplantation and DMBA treatment. Serial sections demonstrate positive co-immunohistochemical staining for GFP (A), desmin (C) and αSMA (E). Boxes outline the same individual cells across the serial sections. Co-immunofluorescence for GFP (B) and αSMA (D) showing a bone marrow derived activated pancreatic stellate cell in the stroma directly adjacent to the PanIN lesion. (TIF) [file pone.0026088.s002.tif]

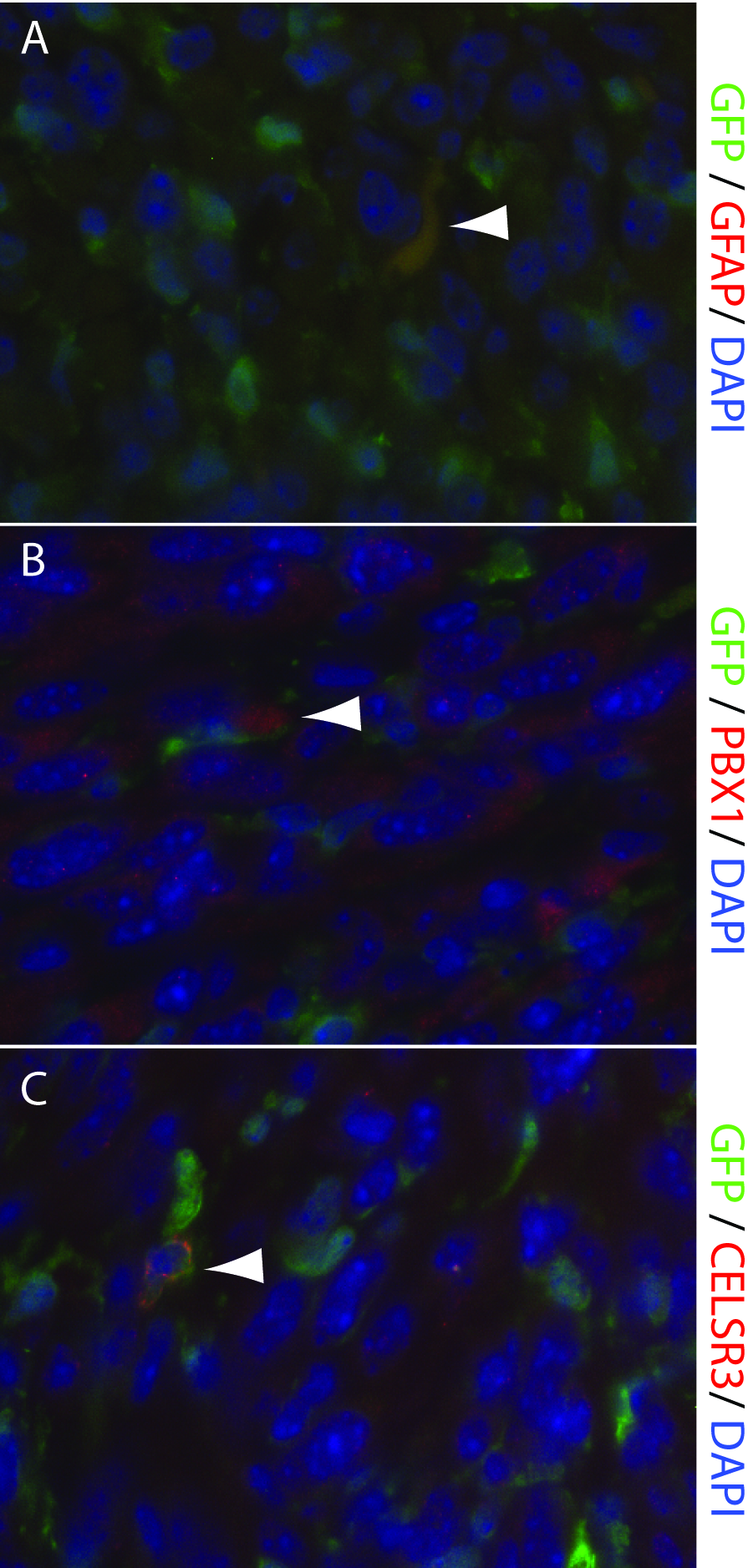

Supplement: Figure S3 — Immunofluorescent characterisation of the bone marrow derived stellate cells within the sarcomatoid-like tumor following treatment with DMBA. Co-immunofluorescence of GFP with (A) glial fibrillary acidic protein (GFAP), (B) pre-B-cell leukemia transcription factor 1 (PBX1), and (C) cadherin EGF LAG seven-pass G-type receptor 3 (CELSR3) to identify bone marrow derived activated stellate cells within the tumor (arrowhead). (TIF) [file pone.0026088.s003.tif]
